# Plasma membrane rafts engaged in T cell signalling: new developments in an old concept

**DOI:** 10.1186/1478-811X-7-21

**Published:** 2009-09-04

**Authors:** Thomas Harder, Dhaval Sangani

**Affiliations:** 1 Sir William Dunn School of Pathology, South Parks Road, University of Oxford, OX1 3RE, UK

## Abstract

Considerable controversy arose over the concept that cholesterol/sphingolipid-rich rafts in the T cell plasma membrane serve as a platform for TCR signalling reactions. This controversy was founded on the initial definition of rafts as detergent resistant membranes which later turned out to misrepresent many features of cell membrane organisation under physiological conditions. Raft-organisation was subsequently studied using a number of detergent-free experimental approaches. The results led to a refined perception of membrane rafts which resolves the controversies. Here we review new biophysical and biochemical data which provide an updated picture of the highly dynamic nanometer-sized cholesterol/sphingolipid-rich raft domains stabilised by protein-networks to form TCR signalling platforms in the T cell plasma membrane.

## Introduction

The T cell antigen receptor (TCR) provides the key signal for activation of T lymphocytes to perform their numerous effector functions in adaptive immune responses. T cells become activated upon engagement of their TCRs by a cognate peptide-MHC ligand presented on the surface of an antigen-presenting or target cell. Early T cell signalling reactions are embedded in the complex and dynamic lipid bilayer matrix of the T cell plasma membrane and are critically defined by their lateral compartmentalisation in plasma membrane domains [1,2]. We will here discuss recent data which provide a detailed picture of the membrane raft characteristics of the plasma membrane domains supporting active TCR signalling protein complexes.

### Protein scaffolds and membrane rafts define TCR signalling plasma membrane domains

The TCR signalling cascade is initiated by phosphorylation of critical tyrosines of the TCR/CD3 complex by the Src-family kinases Lck or Fyn which are anchored in the cytoplasmic leaflet of the T cell plasma membrane. The cytosolic ZAP-70 tyrosine kinase is recruited to the T cell plasma membrane via binding to the tyrosine phosphorylated TCR-complex [3]. ZAP-70 then becomes activated to phosphorylate a defined set of tyrosine residues in the cytoplasmic portion of the trans-plasma membrane adaptor protein Linker for Activation of T cells (LAT) [1,4]. These TCR activation-induced tyrosine phosphorylations of membrane-associated signalling proteins trigger the formation of protein complexes, held together by a cooperative network of protein-protein interactions [5,6]. These complexes assemble into submicron TCR signalling domains in the T cell plasma membrane which were first studied by confocal fluorescence microscopy at the contact zone of a T cell with TCR-activating glass coverslips. These complexes were shown to incorporate numerous cytosolic TCR signalling adaptors and enzymes, driven by TCR activation-induced tyrosine phosphorylations [7]. For a detailed review on TCR signalling microclusters see [8]. Video microscopy technology resolving single molecule movement was employed to monitor the dynamic interaction of membrane-associated signalling proteins with TCR signalling clusters in plasma membrane domains. These studies tracked the movement of Lck and LAT in the T cell plasma membrane and showed retention of these proteins in the submicron TCR signalling domains and their subsequent release. The retention of LAT in the TCR signalling domains depended on the phosphorylation of its tyrosines.

All these reports highlighted protein-protein interactions as critical driving force of TCR signalling complex formation. However specific lipid-mediated interactions at the T cell plasma membrane bilayer are also a central functional element in early TCR signalling. Numerous intracellular signalling proteins interact with plasma membrane lipids of the cytoplasmic leaflet via specific lipid headgroup-binding domains. These interactions and their essential functional consequences for cell surface receptor signalling reactions are excellently reviewed in [9].

The analysis of detergent-resistant T cell membranes had initiated the concept that early TCR signalling steps take place in cholesterol/sphingolipid-rich raft domains of T cell plasma membranes [10] (see Box for an overview of the current perception of raft domains in cell membranes). Confocal fluorescence microscopy was employed to monitor the distribution of TCR signalling plasma membrane sites and to relate their distribution to that of clustered raft markers which show resistance to Triton X 100 detergent solubilisation. This report suggested that the TCR signalling domains represent coalesced raft domains [11]. Moreover, Blue Native Gel Electrophoresis of TCR complexes indicated that the integrity of high molecular weight TCR complexes in T cell plasma membranes depends on membrane cholesterol [12]. However, in consequent studies using Fluorescence Resonance Energy Transfer (FRET), no clustering of generic GPI-anchored raft marker proteins in TCR signalling domains was detected [13]. These studies also showed that an important element of the apparent accumulation of glycosphingolipid raft-marker GM1 at TCR activation sites could be attributed to convolutions of the T cell plasma membrane at these sites which are not resolved by the light microscopical methods used. These and other results formed a basis of well-founded scepticism over the concept that TCR signalling occurs in specific cholesterol/sphingolipid raft domains of the T cell plasma membrane [14,15].

This scepticism was resolved using new methodologies to monitor membrane raft domains in T cells: Accumulation of raft-markers in T cell activation plasma membrane domains was demonstrated by relating fluorescence intensities of fluorescent protein-tagged raft and control non-raft membrane markers. This report indicated that a stabilisation of raft properties at these plasma membrane domains which occurs upon CD28 costimulation [16,17] required the actin-cytoskeleton regulating protein filamin-A [18]. Nanoscale raft organisation in the cytoplasmic and exoplasmic leaflets of T cell plasma membrane was recently also shown by fluorescence correlation spectroscopy [19]. In this study rafts were implicated in phosphatidylinositol-3 kinase dependent signalling reactions downstream of TCR activation [19]. New insights, discussed below, drew a detailed picture of the raft-biophysics and raft-lipid biochemistry of T cell activation plasma membrane domains.

### Plasma membrane domains engaged in TCR activation adopt a specific condensed physical state

**Figure 1:**
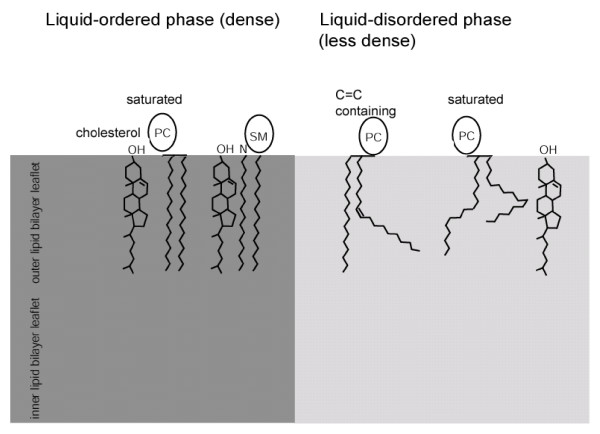
**Liquid-ordered (L_o_) raft phases of the exoplasmic leaflet of the plasma membrane lipid bilayer as modelled in artificial lipid membranes**. Alignment of cholesterol with saturated PC species and SM drives the formation of densely packed liquid-ordered L_o _raft membrane phases. The phases can coexist in model membranes with a less densely packed liquid-disordered (L_d_) membrane phase which is formed by unsaturated phospholipids which do not align tightly with cholesterol.

In an approach to assess physical membrane raft properties, T cell membranes were stained with the fluorescent lipid dye Laurdan, a reporter for the packing density of lipid membranes [20-22], 2-photon microscopy of these fluorescent Laurdan-stained T cells revealed an increase in the hydrophobic packing density of the T cell plasma membrane at TCR activation sites. This was shown to depend on Src-kinase activity, an intact actin cytoskeleton, the presence of LAT, and was inhibited by cholesterol depletion [17]. The condensed state of TCR activation domains correlates with the physical properties of cholesterol/sphingolipid-rich liquid-ordered (L_o_) raft phases which were constituted in artificial membranes composed of mixtures of cholesterol and a saturated phosphatidylcholine (PC) -species or sphingomyelin [23] (Figure 1). The inserted Box outlines the evolution of the lipid raft concept to the current perception of rafts as nanometer-sized highly dynamic cholesterol/sphingolipid domains in cell membranes which are stabilised by protein scaffolds to form membrane platforms for cell biological functions [24].

The incorporation of the oxysterol 7-ketocholesterol (7KC) and the incorporation of the polyunsaturated fatty acid (PUFA) eicosapentanoic acid into T cells perturbed the plasma membrane condensation at TCR activation sites as reported by Laurdan fluorescence [25,26]. PUFAs are incorporated into T cell glycerophospholipids to generate unsaturated phospholipids. Such glycerophospholipids, containing multiple C = C double bonds, disrupt raft phases in model membranes (Figure 1) [26-29]. 7KC likewise causes alteration of ordered membrane phases in artificial liposomes [30,31]. The disruption of plasma membrane condensation at TCR activation sites by incorporation of PUFA and 7KC correlates this membrane structure with the L_o_-lipid phases in model membranes suggesting that such raft lipid-phases comprise a defining element of TCR signalling plasma membrane domains. Importantly, the incorporation of these raft-disrupting lipid compounds into T cell membranes also resulted in an inhibition of several T cell activation parameters [25,27]. 7KC and PUFAs provide possible tools to address the molecular basis of the influence of the condensed membrane state on TCR signalling pathways.

The condensation of TCR activation plasma membrane domains depended on actin cytoskeletal interactions and on the formation of TCR/LAT signalling protein networks [17]. This highlights the protein-interactions as a second important principle which drives the Laurdan fluorescence-reported plasma membrane condensation at TCR signalling sites. Dissecting the crosstalk of protein- and lipid-mediated principles which drive this raft coalescence requires new technologies to allow live tracking of the condensation of TCR-signalling domains in T cell plasma membranes at a nanometer-scale resolution.

## Box

### The evolving concept of cholesterol/sphingolipid raft domains

The analysis of polarised lipid sorting in epithelial cells sparked the initial proposal that sphingolipid and cholesterol clusters (rafts) form platforms for the generation of intracellular transport vesicles to the apical plasma membrane [32]. It was then reported that on entering this polarised intracellular transport route to the apical plasma membrane of epithelial cells, GPI membrane-anchored PLAP protein acquires resistance to solubilisation by Triton X-100 at 4°C [33]. Plasma membrane-anchored glycolipids, GPI-anchored proteins, and protein-kinases were found in lipid/protein complexes resisting similar mild conditions of detergent-solubilisation. Consequently specific cell surface signalling functions were also proposed to occur in sphingolipid-rich plasma membrane complexes [34]. These observations led to the hypothesis that raft domains form functional platforms for numerous membrane-associated cellular activities [35]. In the following we will outline the developments which led to the current perception of membrane rafts and include new data which provide support and new details of rafts in cells as defined at the Keystone Symposium on Lipid Rafts and Cell Function in 2006 [36].

### In vitro reconstitution of liquid-ordered raft-phases

The lipid environment which conferred Triton X-100 insolubility to the GPI-anchored protein PLAP was characterised by reconstituting PLAP into liposomes [37]. In such model membrane systems densely packed liquid-ordered (L_o_) membrane phases formed by cholesterol and sphingolipid or saturated PC (Figure 1) indeed fulfilled the raft-criterion of Triton X-100 insolubility [23,38]. This was the basis of the important assumption that these L_o _phases represent *in vitro *models of rafts in cells. It was demonstrated in artificial membranes composed of ternary lipid mixtures that these L_o _phases can coexist with a liquid-disordered (L_d_) phase which is formed by unsaturated PC and readily solubilised by detergent [28,38](Figure 1). However, it was demonstrated in artificial membranes that detergent-treatment induced the formation of raft-like domains in membranes not present prior to the addition of the detergent [39]. Consequently it is not possible to equate detergent resistant membrane (DRM)-association with an *in situ *organisation of cellular membrane raft components. This observation resulted in considerable controversies over the structure, function and even the existence of rafts in cells under physiological conditions [14]. These were resolved using novel experimental technologies which revealed important new insights leading to the current perception of sphingolipid/cholesterol-rich membrane raft domains.

### Characterisation of dynamic nanometer-scale raft-protein and raft-lipid clusters in cells

A major step in the evolution of the concept of rafts in cell membranes was the characterisation of small raft-protein clusters and observation of transient anchoring of raft-lipids in nanometer-sized cholesterol-dependent plasma membrane domains. Initial Fluorescence Resonance Energy Transfer (FRET) approaches showed that a fluorescently-labelled GPI-anchored raft reporter formed small cholesterol-dependent clusters in cells [40]. Similar small clusters were described for a DRM-associated raft protein anchored by fatty acyl-modification in the cytoplasmic leaflet of the plasma membrane [41,42]. Later studies showed that the majority of a GPI-anchored fluorescent raft reporter protein was actually detected as monomers but a 20-40% fraction resides in clusters with sizes of ≥5 nm diameter which generally contain 2, maximally 4, reporter molecules [43,44]. These nanometer-sized clusters of GPI-anchored raft markers were shown to be relatively immobile and their cholesterol-dependent clustering requires anchoring to the cortical actin cytoskeleton [44]. Stimulated Emission Depletion (STED) nanoscopy was employed to compare transit times of single molecules of a GPI-anchored raft-protein or a raft-sphingolipid in comparison with a phosphatidylethanolamine non-raft lipid through a ~50 nm focal area on the plasma membrane. This showed a cholesterol-dependent and short-lived (in the order of 10-20 ms) trapping of the lipid raft-probes in immobile domains which dwell in plasma membrane areas of < 20 nm diameter [45]. These analyses of plasma membrane organisation led to the definition of highly dynamic raft domains in the cell plasma membrane which have a diameter of few nanometers and are thus not resolvable by conventional light microscopy.

### Stabilisation and coalescence of plasma membrane rafts to form micrometer-scale domains upon lateral crosslinking of raft-components

The even distribution of raft markers at the cell surface as it appears in light microscopy is dramatically altered upon crosslinking of raft proteins and lipids. Different DRM-associated lipid- or protein-raft markers were shown to co-cluster in the same plasma membrane domains if independently crosslinked. These raft-patches were separated from clusters of crosslinked non-raft plasma membrane proteins suggesting that patches of clustered raft-markers form by coalescence of raft domains following crosslinking of raft membrane components [46].

This concept received strong recent support by experiments in which raft glycosphingolipid GM1 in lipid membranes was crosslinked using the pentavalent choleratoxin B subunit (CTB). In model liposomes in a single liquid phase, which is close to the point of separation into L_o_/L_d _phases, the crosslinking of GM1 by CTB induces segregation of L_o _and L_d _membrane phases visible by light microscopy [47].

A CTB-mediated GM1 crosslinking also triggered the segregation of micrometer-sized GM1-domains at 37°C in large (>10 μm) plasma membrane-derived spheres from A431 epidermoid carcinoma cells [48]. The GM1/raft-domains of these plasma membrane-derived spheres excluded the non-raft membrane protein transferrin-receptor and concentrated different DRM-defined raft membrane proteins: the single-pass transmembrane protein LAT, VIP17 membrane tetra-spanning protein, and GPI-, and fatty acyl-anchored proteins. Thus raft and non-raft marker membrane proteins exhibited the expected inclusion and exclusion into or from the coalesced raft domains of these plasma membrane-derived spheres [48]. In summary, the metastable nanometer-sized rafts as characterised in resting cells are stabilised by crosslinking of raft components and can then coalesce into functional micrometer membrane domains to form functional platforms for cell biological activities.

## End Box

### The molecular lipid composition of TCR signalling plasma membrane domains

New mass spectrometric methodology was employed to characterise the molecular raft lipid composition of TCR signalling plasma membrane domains. These T cell plasma membrane domains were immunoisolated as native (not detergent-treated) plasma membrane fragments [49] using TCR-activating magnetic beads which were conjugated to Jurkat T leukemic cells. These conjugates were homogenised mechanically and native Jurkat plasma membrane fragments bound to the magnetic beads were isolated. The molecular lipid composition of these T cell plasma membrane fragments was quantitatively charted using a mass spectrometry program developed for comprehensive characterisation of membrane lipidomes [50,51]. Comparison of the molecular lipid composition of these isolated TCR signalling plasma membrane domains with that of immunoisolated control plasma membrane fragments provided the first direct evidence for a lateral segregation of specific molecular lipid species into plasma membrane domains [26].

TCR signalling plasma membrane domains accumulate cholesterol, sphingomyelin, and saturated phosphatidylcholine species. In model membranes a mixture of these lipids form L_o _phases akin to cell membrane rafts [23] (Figure 1, see Box for an outline of the evolving lipid raft concept as it stands today). Therefore, TCR signalling plasma membrane domains are characterised by the physical condensed raft structure and an accumulation of raft (L_o _phase) forming lipid species. This coalescence and stabilisation of rafts at TCR signalling platforms most likely result from the signalling-induced crosslinking and immobilisation of raft- membrane proteins at TCR activation domains [52].

The molecular mechanisms of coupling between the exoplasmic and the cytoplasmic plasma membrane bilayer leaflets at lipid raft domains is matter of intense current interest as it is central to the signal transduction via raft domains. Patches of crosslinked raft markers anchored in the exoplasmic plasma membrane leaflet were shown to colocalise with inner leaflet raft-associated Lck [11]. This coupling of inner and outer plasma membrane leaflets at coalesced rafts was later confirmed using an Lck-membrane anchored fluorescent protein as an inner plasma membrane leaflet raft reporter [53].

The two leaflets of plasma membrane lipid bilayer display a marked lipid asymmetry [54,55]. The exoplasmic leaflet of the plasma membrane contains the bulk of the sphingomyelin (SM) and phosphatidylcholine (PC) whereas the cytoplasmic plasma membrane leaflet strongly enriches phosphatidylethanolamine (PE), phosphatidylserine (PS), and phosphatidylinositol (PI). The characteristic raft-defining lipid composition of TCR activation domains concerns specific PC species and SM which are concentrated in the exoplasmic plasma membrane leaflet (Figure 1). The inner leaflet-preferring phospholipids of the Jurkat T cell plasma membrane bilayer contain very few fully saturated phospholipid species and the TCR signalling domains showed no obvious preference for more saturated inner leaflet phospholipids species. This indicates that organization of the inner and outer plasma membrane leaflet at TCR signalling sites are different. Nevertheless the characteristic molecular lipid composition of these domains extends to PE, PI, and PS enriched in the cytoplasmic plasma membrane leaflet suggesting some sort of coupling between the lipid bilayer leaflets at rafts [26]:

Importantly, total PE was found not to accumulate in active plasma membrane TCR signalling domains. Hence, the lipidome of TCR activation plasma membrane domains does not confirm the proposed general accumulation of PE at TCR activation raft domains [56]. However, the relative fraction of plasmenyl PE species, which are characterised by an ether-bound hydrocarbon chain, is significantly increased over plasma membrane not engaged in TCR signal transduction. The increased fraction of plasmenyl PE species was likewise observed in the lipidomes of other raft membranes prepared by detergent-free methods [57,58].

The inner leaflet lipids the TCR signalling plasma membrane domains moreover significantly accumulate the negatively charged phospholipid PS, however with no obvious preference for a particular species. PUFA-treatment resulted in loss of PS enrichment in the TCR signalling plasma membrane domains [26] correlating the raft-like membrane condensation at these plasma membrane domains with PS accumulation at their cytoplasmic leaflet. Specific functional interactions of PS-rich membranes with the TCR/CD3 complex and the TCR signalling machinery have been reported in a series of *in vitro *studies using artificial lipid membranes [59-62]. The membrane anchors of key signalling proteins, members of Ras superfamily or the Src kinase, contain stretches of basic amino acids. In fluorescence microscopy studies the net positive charge of these membrane-targeting regions correlates with the localisation of these proteins at PS-rich cell membranes suggesting an anchoring of these proteins to the PS-enriched membranes via electrostatic interactions [63]. All these data strongly suggest a functional role of PS accumulation for different steps of TCR signalling.

### Lateral membrane compartmentalisation of Ras signalling

The activation of signalling pathways downstream of Ras small GTPase provides a crucial element of signal transduction by numerous tyrosine kinase-controlled cell surface receptors, including the TCR. Signalling functions of Ras critically depend on their anchoring in the cytoplasmic leaflet of the plasma membrane by lipid-modifications [64]. It was shown in non-hematopoietic cells that lateral compartmentalisation of the 3 Ras isoforms in plasma membrane domains is a key determinant in their function. An average of 7 molecules of a specific isoform of membrane-anchored Ras form cluster in domains of 10-20 nm diameter by mechanisms which rely on specific protein scaffolds and on cholesterol-dependent mechanisms [2,41]. Recent computer-based *in silico *models show the profound consequences of Ras-anchorage and raft-lipid dependent Ras-clustering in plasma membrane domains for Ras-downstream signalling [2,65]. Ras clusters in non-hematopoietic cells were modelled as digital "nanoswitches" for activation of the MAP kinase pathway in response to a signal via cell surface receptors. The digital signals given by estimated 40000 of these nanoswitches in the plasma membrane of these mammalian cells were modelled to integrate into a high-fidelity cellular response matching a graded receptor stimulus [64,66].

The organisation of Ras in plasma membranes of hematopoietic cells has not yet been characterised. In B- and T-lymphocytes the activation of a small number of the respective B cell antigen receptor (BCR) or T cell antigen receptors (TCR) triggers a decisive (digital) activation response of the MAP kinase pathway, once a certain threshold number of receptors activated is passed [65]. Ras is activated by an exchange of Ras-bound nucleotide GDP by GTP. The formation of the active Ras is mediated by Ras GDP/GTP exchange factors (RasGEFs) which are recruited to cell membranes. In B- and T-cells the activities of two RasGEFs; RasGRP and SOS, critically cooperate at the lymphocyte plasma membrane to mediate Ras activation in response to antigen receptor activation [67]. Recent reports combined *in silico *and *in vitro *analyses to model the sequential activity of these two RasGEFs to activate Ras. For SOS the binding of Ras to a non-catalytic allosteric site stimulates its RasGEF activity. Activation of SOS via this allosteric binding site has a strong preference for the active RasGTP [68]. The preferential activation of SOS' GEF activity by its enzymatic RasGTP reaction product provides a critical positive feedback loop which drives a digital SOS GEF activity circuit. Ras activation by RasGRP, on the other hand, follows a sequence of signalling steps which provide a graded (analogue) downstream response following BCR or TCR activation. The Ras GEF activity of RasGRP provides an initial Ras-activation response to TCR or BCR signal which ignites the positive feedback loop of SOS' RasGEF activity to support a digital Ras activation circuit [65]. It is tempting to speculate that the raft lipid- and protein-dependent clustering of Ras molecules in membrane domains, as reviewed above, connects allosteric binding of SOS to RasGTP and simultaneous catalysis of GDP/GTP exchange on a neighbouring Ras to eventually trigger the activation of all Ras molecules in the cluster.

## Conclusion and future outlook

Early TCR signalling reactions take place in domains of the T cell plasma membrane. New data drew a detailed picture of the biophysical raft structure and the complex raft lipid biochemistry of TCR signalling domains. *In vitro *reconstitution of lipid membrane-based TCR/LAT early signalling machineries on defined artificial lipid membranes [62] may pave the way to unravel the influence of the lipid bilayer platform on the activity of such lipid/signalling protein complexes.

*In silico *modelling of signalling downstream of Ras nanoclusters in plasma membrane raft domains in non-hematopoietic cells provided a first description of crucial signalling mechanisms determined by raft domains. Such systems biology approaches [65,66] provide a further outlook on new methodology deciphering the role of lipid membrane platform in cell surface receptor signalling.

## Abbreviations

7KC: 7-ketocholesterol; BCR: B cell antigen receptor; DRM: detergent resistant membranes; FRET: fluorescence resonance energy transfer; RasGEF: GDP/GTP exchange factor for Ras; GPI: glycosylphosphatidylinositol; LAT: linker for activation of T cells; L_o_: liquid-ordered; L_d_: liquid-disordered; PUFA: polyunsaturated fatty acid; STED: stimulated emission depletion; TCR: T cell antigen receptor.

## Competing interests

The authors declare that they have no competing interests.

## Authors' contributions

TH and DS outlined the framework and wrote this review. Both authors approve the final manuscript.
